# Immunometabolism in HIV Reservoirs: Implications for Latency and Comorbidities

**DOI:** 10.3390/v18080813

**Published:** 2026-07-24

**Authors:** Mary-Elizabeth Zipparo, Rebecca T. Veenhuis

**Affiliations:** 1 Department of Molecular and Comparative Pathobiology, Johns Hopkins University School of Medicine, Baltimore, MD 21205, USA; mzippar1@jhmi.edu; 2 Department of Neurology, Johns Hopkins University School of Medicine, Baltimore, MD 21205, USA

**Keywords:** immunometabolism, CD4 T cells, monocytes, macrophages, HIV, latency, reservoir, comorbidities

## Abstract

Compelling research has consistently demonstrated a strong relationship between immunometabolism and infectious disease, including the ways in which viral infections alter the metabolic state of immune cells to promote survival. Human immunodeficiency virus (HIV) has been particularly noted for its ability to reprogram the metabolism of cells that contribute to viral persistence. The purpose of this review is to summarize current knowledge of the metabolic state of CD4 T cells and myeloid cells (monocytes/macrophages), two of the primary cell types targeted by HIV. The studies discussed reveal distinct metabolic profiles in both cell types during initial infection, active replication, and latency. In addition, we examine how these metabolic alterations may contribute to the increased frequency and severity of comorbidities observed in people with HIV (PWH). Understanding the impact of HIV infection and latency on immunometabolism may provide deeper insight into long-term viral persistence and support the identification of novel therapeutic targets to reduce chronic inflammation and inform future cure strategies for PWH.

## 1. Introduction

Human immunodeficiency virus (HIV) continues to be a global epidemic, even with effective antiretroviral therapy (ART) [1]. While ART can control viral replication and prevent transmission, the latent reservoir is the primary barrier to a cure [1]. The HIV reservoir persists in CD4+ immune cells, notably CD4 T cells and myeloid cells (monocytes/macrophages) [1,2]. Recent evidence demonstrates that HIV primarily infects metabolically active immune cells, regardless of activation status, and the natural transition to a memory and quiescent state allows for the establishment of the latent reservoir [3,4]. Transcriptional silencing of the provirus allows the reservoir to persist in cells while evading immune detection [5]. Even with the virus in a latent state, people with HIV (PWH) still experience higher risks of HIV-related comorbidities, including cognitive impairment, type 2 diabetes, cancer, and cardiovascular disease [6,7].

Immunometabolism has become a recent focus of the scientific community. Compelling research is consistently published demonstrating a strong relationship between immunometabolism and infectious disease state, and how viral infections alter the immunometabolic state of immune cells for their own survival in the host [8,9]. HIV has been specifically highlighted for its ability to hijack the immunometabolic state of reservoir-containing cells, which likely contributes to the increase in severity of various comorbidities experienced by PWH [10,11]. The purpose of this review is to highlight the effect of HIV infection and latency on immunometabolism, and how it may further exacerbate HIV-related comorbidities.

## 2. Immune Cell Metabolism

This review will focus on metabolism in immune cells that compose the majority of the HIV reservoir: CD4 T cells, monocytes, and macrophages. However, it is important to note that other immune cells, including CD8 T cells, dendritic cells, and natural killer (NK) cells, also experience alterations in metabolism upon HIV infection [12,13,14,15,16,17,18]. These cell types have been previously discussed and are not within the scope of this review [8,10].

### 2.1. CD4 T Cells

CD4 T cells exhibit substantial plasticity when it comes to their metabolic state [8,19,20]. Naïve T Cells (T_n_) are the most metabolically quiescent of the CD4 subsets [21]. T_n_ cells rely primarily on mitochondrial oxidative phosphorylation (OXPHOS) for their energetic needs, with lower levels of glucose uptake required for glycolytic activity [21]. Major histocompatibility complex (MHC) engagements with the T cell receptor (TCR) and stimulation with interleukin (IL)-7 are necessary to prevent T_n_ cell death [21,22]. Upon encountering their cognate antigen, T_n_ cells will take on an effector T cell (T_ef_) phenotype [20,21]. The interaction of the TCR and antigenic peptide presented by MHC, as well as costimulatory receptor CD28 with CD80/CD86 on the antigen-presenting cell, initiates a cascade of intercellular signaling, specifically engaging the mechanistic Target of Rapamycin (mTOR), phosphoinositide 3-kinase (PI3K)/AKT pathways [20,21]. These conditions, and the presence of IL-2, promote T_ef_ cell differentiation and expansion, along with substantial metabolic alterations to promote these changes [20,21].

T_ef_ cells primarily rely on glycolysis, as well as glutaminolysis, to meet their energetic requirements for effector function and proliferation [20,21,23]. Similar to the “Warburg Effect” seen in highly proliferative cancer cells, activated T_ef_ cells favor aerobic glycolysis over other metabolic pathways [21,23]. This effect leads to an accumulation of glycolytic metabolites that can also enter alternative pathways, such as the Pentose Phosphate Pathway (PPP) for Nicotinamide Adenine Dinucleotide Phosphate (NADPH) and nucleotide production [23]. Adenosine Triphosphate (ATP) is also heavily utilized, and T_ef_ cells typically have higher levels of mitochondrial biogenesis to meet these needs, partially regulated by AMP-activated protein kinase (AMPK) sensors [20,21,23]. Fatty acid synthesis (FAS) is also upregulated to promote T cell differentiation and provide “building blocks” for expansion and proliferation [24]. Differentiation of T_ef_ cells into T helper (Th)1, Th2, Th17, and T regulatory (reg) phenotypes from environmental cues also promotes distinct metabolic pathways depending on the subset [20,21]. In general, all pro-inflammatory phenotypes tend to have higher levels of glycolysis and glucose intake, while T_reg_ rely on OXPHOS and fatty acid oxidation (FAO) [24,25]. As a result, T_reg_ have higher mitochondrial mass, but lower levels of glucose-1-transporter (GLUT1) compared to the pro-inflammatory phenotypes [25,26,27]. The master transcription factor characteristically expressed in T_reg_, Forkhead box P3 (FOXP3), helps to actively shape metabolism by suppressing glycolysis and enhancing OXPHOS [20,25].

Memory T cells (T_m_), similar to T_n_ and T_reg_, rely on OXPHOS and FAO [21,24]. However, T_m_ cells will maintain a higher level of metabolic activity compared to T_n_ cells [21]. T_m_ cells experience moderate to low levels of GLUT1 expression compared to T_n_ cells [24]. This expression of GLUT1, paired with elevated FAO levels, allows for a “primed” metabolic state should they encounter their cognate antigen in future settings [4,21,24]. T_m_ cells also have larger mitochondria, which allows for a higher respiratory capacity [21].

### 2.2. Myeloid Cells

Monocytes, a subset of myeloid cells, are composed of three major subsets. The first subset, and most abundant in circulation, are classical monocytes [28]. Classical monocytes, characterized by high CD14 and no CD16 expression, rely primarily on glucose, utilizing the glycolytic pathway and PPP [28,29,30]. Non-classical monocytes, the second most abundant with low CD14 and high CD16 expression, utilize FAO, have higher levels of mitochondrial activity, and therefore rely on OXPHOS pathways for their energetic requirements [28,29,31]. Intermediate monocytes, which are the least abundant and express both CD14 and CD16, have been the least well documented regarding their metabolic processes [28]. While this monocyte subset experiences an increase in gene expression related to antigen processing and presentation, their metabolism alters as they transition from classical to non-classical phenotypes, with no real enrichment in any one specific pathway [29]. Due to the short-lived nature of monocytes in circulation, understanding their metabolic requirements can be relatively challenging.

Monocytes quickly migrate out of the blood and into tissue, where they differentiate into unique macrophage phenotypes depending on tissue context [32]. Macrophage phenotypes, like T cells, have vastly different metabolic states [33,34,35,36]. Upon Toll-Like Receptor (TLR) stimulation in an inflammatory environment, macrophages adopt a proinflammatory (M1) phenotype and a similar metabolic profile to T_ef_ cells [35]. M1 macrophages experience a significant increase in glucose uptake and glycolytic metabolism. Under pro-inflammatory conditions, macrophages favor aerobic glycolysis for swift ATP generation, like the “Warburg Effect” described above [35]. As a result of glycolytic intermediate accumulation, the PPP is also upregulated to promote NADPH production for nucleotide biosynthesis, as well as nitric oxide (NO) and reactive oxygen species (ROS) production to contribute to pathogen clearance [33,35]. OXPHOS is also inhibited and significantly downregulated via two breaks in the tricarboxylic acid (TCA) cycle [33]. One break leads to the downregulation of isocitrate dehydrogenase (IDH), resulting in an accumulation of citrate. This promotes FAS for membrane integrity and increases NO and ROS accumulation via itaconate accumulation [33,34,35]. FAS is favored over FAO in M1 macrophages, with enhanced fatty acid uptake and synthesis from endogenous lipids, aiding in macrophage activation and structural support [33,34,35]. However, it is important to note that FAO is still utilized to support certain inflammatory functions, such as the inflammasome, in M1 macrophages [37]. The second break causes succinate to accumulate, which boosts mitochondrial ROS (mtROS) for pathogen clearance and hypoxia-induced factor 1-alpha (HIF-1α) stabilization for pro-inflammatory cytokine secretion as well as inflammasome activation [33,34,35,37]. These combined metabolic factors promote M1 polarization.

During the resolution phase of infection, environmental cues such as IL-4 and IL-13 promote an anti-inflammatory (M2) macrophage phenotype. M2 macrophages have a unique metabolic profile compared to their M1 counterparts [33,34,36,37]. M2 macrophages heavily rely on FAO, glutamine, and OXPHOS, with functional TCA cycles for their steady-state metabolic requirements [33,34,35,36,38]. However, some studies have shown that blocking or inhibiting FAO in M2 macrophages does not impact M2 polarization [35]. M2 macrophages are also characterized by high arginase-1 expression, increasing ornithine and polyamine biosynthesis pathways for cell growth and proliferation [35]. The majority of glucose taken up by M2 macrophages goes into cellular respiration, fueling OXPHOS as opposed to glycolysis and other glucose-requiring pathways [33]. M2 macrophages also accumulate the metabolic intermediate α-ketoglutarate (α-KG), which promotes M2 polarization by supporting the TCA cycle and inhibiting pro-inflammatory cytokine production via metabolic and epigenetic alterations [36,38]. While macrophage polarization is a spectrum, not a dichotomous categorization of M1 and M2, it is clear that the metabolic state contributes to the polarization and functional state of this cell type [33,34,36,37].

## 3. Immunometabolism and HIV in CD4 T Cells

### 3.1. Initial Infection and Active Replication

**Figure 1:**
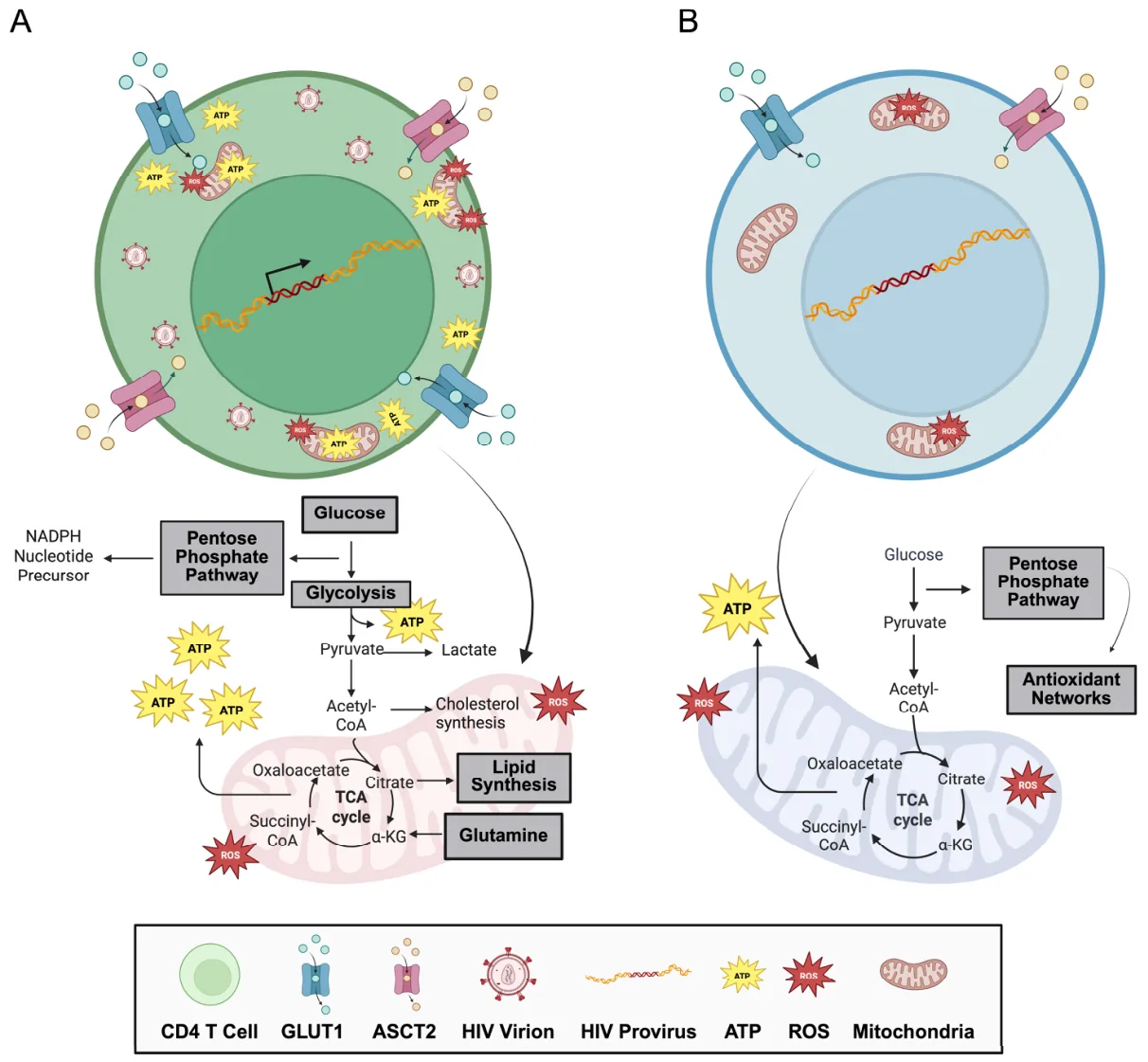
CD4 T cell metabolism during active and latent HIV infection. (**A**) Active HIV infection is reflected by increased GLUT1 and ASCT2 expression on CD4 T cell surface. This results in increased glycolysis and glutaminolysis to provide energy for viral production and replication. In the metabolically active mitochondria (red), HIV infection results in increased glucose consumption for both the glycolytic pathway and pentose phosphate pathway (PPP) for energy and nucleotide synthesis. TCA intermediates are also fueled via glutamine uptake and can be used for fatty acid synthesis, which is required for viral budding. HIV infection also results in increased oxidative stress in the CD4 T cell. (**B**) Latently infected CD4 T cells are more metabolically quiescent compared to actively infected CD4 T cells, as demonstrated by the blue mitochondria. They experience a decrease in glycolytic activity with lower expression of GLUT1 and ASCT2, which is thought to be induced by the transition to memory state and viral latency. Glucose is shunted to the PPP, which promotes engagement of various antioxidant networks that combat oxidative stress. In the quiescent state, latently infected memory cells will also rely on OXPHOS, as is seen in memory CD4 T cells. Created in BioRender. Zipparo, M. (2026) https://BioRender.com/rut0mle.

CD4 T cells are one of the primary targets for HIV, and initial infection, active replication and latency all result in distinct metabolic phenotypes in this cell type (Figure 1). However, not all CD4 subsets are equally likely to be targeted by the virus, with effector subsets being the most, and the naïve subset among the least, permissive to infection [3,10]. It was widely reported that the activation state of CD4 T cells is associated with infectability, yet recent studies have shown that metabolic state is more important for determining permissiveness of infection, independent of activation state [3,4]. Once HIV has infected a cell, it will utilize factors from the host to produce infectious virions, which requires a greater metabolic demand [8,39,40,41,42]. One of the primary sources of energy HIV can utilize via the host cell is glucose, and many studies have shown both in vitro and in vivo that HIV preferentially infects CD4 T cells with high levels of GLUT1 and glycolysis [3,42,43,44]. In response to acute infection, CD4 T cells upregulate GLUT1, which allows for increased glucose uptake, glycolytic activity and cellular activation [4,42]. One study reported significantly elevated levels of GLUT1+ CD4 T cells among treatment-naïve PWH compared to healthy individuals. This was also correlated to higher levels of activation and glucose uptake. An inverse correlation was also observed with GLUT1-expressing CD4 T cells and absolute CD4 T cell count [44]. Treatment-naïve PWH also experience hypermetabolic responses, with elevated p-Akt levels both prior to and in response to stimuli [43]. Higher levels of hexokinase activity, specifically HK1, in infected cells have also been observed and coincide with active viral replication [42]. Other studies that have highlighted the importance of glycolysis for HIV infection and shown that cells treated with glycolytic inhibitors, such as 2-deoxy-D-glucose (2-DG), impair the ability of HIV to successfully infect a CD4 T cell [3,39]. Amino acids, such as glutamine, are another excellent source of energy utilized by CD4 T cells, and the Alanine, Serine, Cysteine Transporter 2 (ASCT2) is shown to be expressed in subsets that are more susceptible to HIV [10,45,46,47]. Recent work has suggested that increased glutamine uptake can be used to fuel OXPHOS and α-KG production, supporting HIV infection and reverse transcription in CD4 T cells [10,46,47]. Similar to blocking glycolysis, blocking glutamine metabolism with the glutamine analog 6-diazo-5-oxo-L-norleucine (DON) impairs HIV replication, suggesting its importance in facilitating infection [3].

While the importance of glycolysis and GLUT1 expression has been heavily focused on in the literature, it is important to note that CD4 T cells experience an overall metabolic reprogramming with HIV infection [3,40]. While important at the beginning stage of infection and replication for ATP output, HIV infection of CD4 T cells is ultimately known to disrupt OXPHOS, favoring glycolysis for energetic requirements and FAS for viral budding [39,40,48]. Accessory proteins of the virus can directly disrupt mitochondrial-associated ER membranes (MAMs), which further induces oxidative stress to the cell and promotes metabolic reprogramming [48,49]. Together, this results in increased metabolic dysregulation and chronic inflammation of infected CD4 T cells, characterized by mtDNA synthesis and enhanced ROS production [4,48,50]. The stress and damage done to mitochondria can also result in increased apoptotic cell death [50]. In addition, the PPP is utilized to increase the dNTP pool needed for viral replication and NADPH to help with ROS production and oxidative stress [10,40].

### 3.2. ART-Suppressed Infection

As mentioned above, while HIV replication and infection occur in metabolically active cells, latent HIV is typically found in memory T cell subsets, where the energetic needs are different [3,10]. In general, latently infected CD4 T cells, mostly T_m_, are less metabolically active, but maintain a unique metabolic signature compared to uninfected CD4 T cells [3,51]. One of the largest shifts in metabolism during viral suppression, as observed in primary CD4 T cells and latently infected Jurkat cell lines, is the downregulation of glycolysis, which is correlated with decreased levels of the enzymes involved in the glycolytic pathway, such as glucose-6-phosphate isomerase (GPI) [51]. GPI commits metabolites to the glycolytic pathway, rather than the alternative PPP. As a result, latently infected cells have been shown to rely on NADPH, a product of PPP, which can help increase antioxidant networks to combat oxidative stress and ROS-mediated apoptosis [51,52,53]. Importantly, inhibiting the PPP and antioxidant pathways and upregulating the glycolytic pathway results in the reactivation of latent HIV [51]. Interestingly, a different study demonstrated that virally suppressed PWH maintained elevated GLUT1 levels compared to healthy controls in multiple CD4 T cell subsets, with increased glucose uptake. GLUT1+CD4+ T cells in the same cohort of individuals also maintained elevated activation markers (HLA-DR and CD38) [44]. A different study utilizing a cohort of virally suppressed (vs)PWH reported elevated levels of p-AKT, a downstream effector of PI3K, compared to uninfected controls, along with hypermetabolic responses to stimulation with anti-CD3/CD28 microbeads. This pathway is heavily involved in GLUT1 translocation to the cell surface membrane, which was also observed in vsPWH [43]. Treatment of CD4 T cells from vsPWH with 2-DG following TCR stimulation inhibited HIV amplification from latently infected CD4 T cells [3]. Together, this work highlights the importance of glucose metabolism in viral suppression, as well as the potential for elevated and accelerated metabolic responses compared to people without HIV (PWoH).

mTOR, a master regulatory protein that controls metabolism, cell growth, and survival, is tightly regulated with T cell differentiation and consequently linked to HIV infection and latency [10,40,54,55,56]. The mTOR pathway is upregulated by CD4 T cell activation and metabolites, such as α-KG from glutamine metabolism, and is downstream of the PI3K/Akt pathway [10,40,57]. mTOR activation has been shown to increase CD4 T cell susceptibility to HIV infection and HIV replication [40,54,56]. Previous research highlighted how metabolic pathways directly linked to mTOR signaling, or lack of signaling via inhibition of mTOR, can alter the epigenetic landscape and both tat-dependent and tat-independent transcription of HIV-infected CD4 T cells, ultimately contributing to the latent state of the virus [10,40,55]. Modifications made to the chromatin are tightly linked to cellular metabolites, including Acetyl-CoA and one-carbon metabolites, which have been shown to impact the availability of the HIV provirus LTR promoters [40,58].

While ART is a critical lifesaving medication for PWH, it has been shown to result in metabolic dysfunction [54,59,60,61]. Different classes of ART drugs, particularly nucleoside/nucleotide reverse transcriptase inhibitors (NRTIs), non-nucleoside reverse transcriptase inhibitors (NNRTIs), and protease inhibitors (PIs), are known to cause mitochondrial dysfunction, causing issues including dyslipidemia, lipodystrophy, altered mtDNA synthesis, increased ROS production, and reduced electron transport chain (ETC) function [48,50,54,61]. A recent review discussed how the activation of inflammatory pathways was associated with these changes in metabolism, potentially contributing to sustained chronic inflammation and elevated markers of immune exhaustion commonly found in PWH [54]. In addition, other studies have shown that ART does not normalize the metabolic reprogramming that occurs following HIV infection. Analysis of serum from a cohort of vsPWH demonstrated that the levels of metabolites associated with glycolysis, the PPP, lactate production, and β-oxidation of long-chain fatty acids did not return to normal values, as compared to matched controls without HIV [59]. The persistent shift in metabolic demands within CD4 T cells from PWH may also contribute to chronic immune activation observed despite successful viral suppression [50,54,59].

## 4. Immunometabolism and HIV in Myeloid Cells

### 4.1. Initial Infection and Active Replication

**Figure 2:**
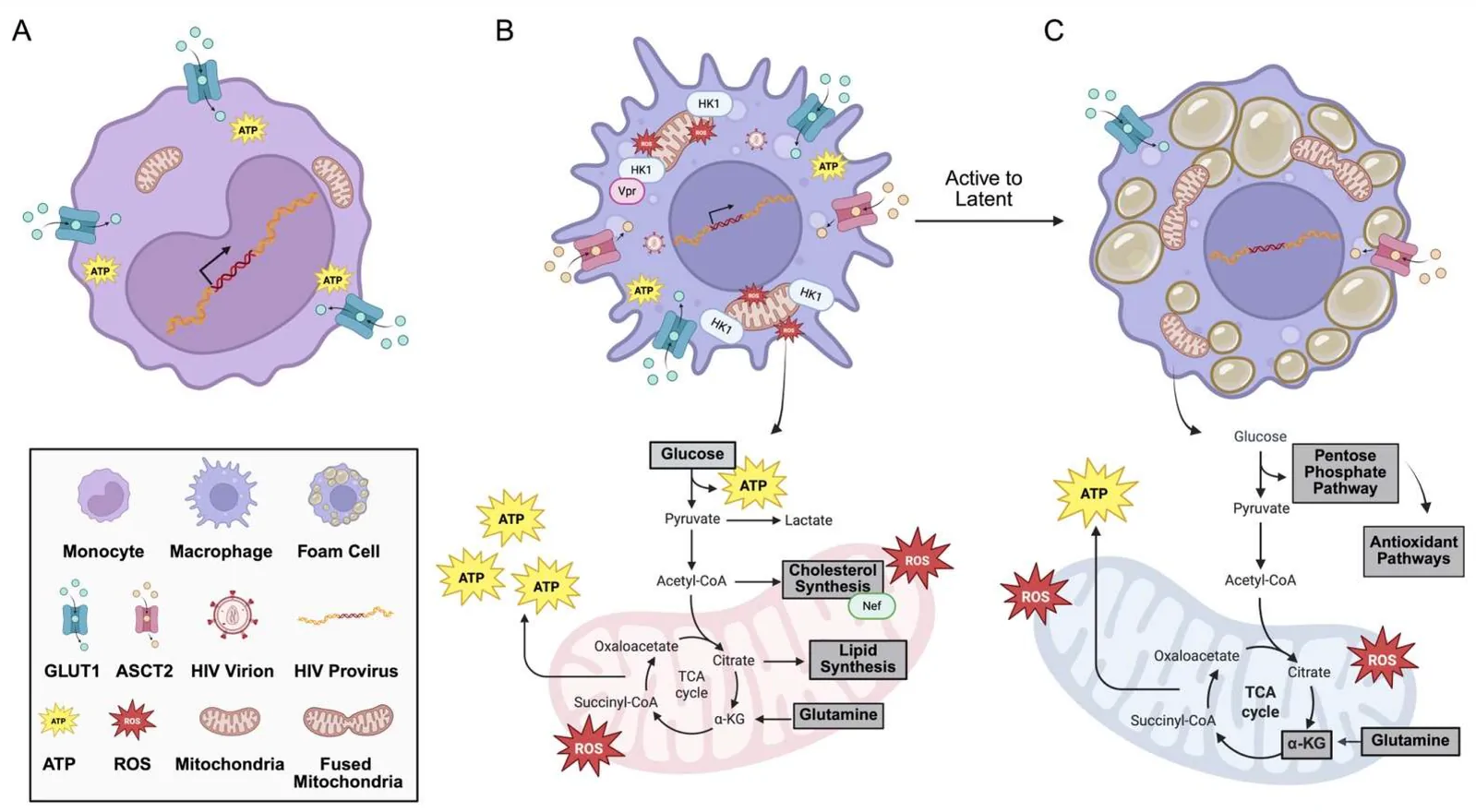
Monocyte and macrophage metabolism during active and latent HIV infection. (**A**) HIV-infected monocytes experience an increase in GLUT1 expression, as well as glucose uptake. Levels of GLUT1 remain elevated following ART initiation compared to PWoH, but lower than treatment-naïve PWH. (**B**) Macrophages undergoing active HIV infection and replication, like CD4 T cells, experience an increase in metabolic activity, including upregulated glycolytic enzymes to promote macrophage survival. HK1 associates with the mitochondrial membrane, as facilitated by Vpr, for this exact reason. These macrophages are characterized by an increase in glucose uptake, glutamine uptake, and dysregulated lipid synthesis/metabolism. This dysregulated cholesterol synthesis and storage is partially due to Nef disrupting the cholesterol efflux pump ABCA1. (**C**) Latently infected macrophages are characterized by increased mitochondrial fusion, dysregulated OXPHOS, and lipid droplet accumulation forming a foam cell phenotype. These macrophages also utilize glutamine as an alternative energy source, in addition to glucose. Glutamine can provide precursors, including α-KG, to help fuel the TCA cycle. Glucose can be shunted into the PPP to help with oxidative stress by increasing antioxidant networks. Created in BioRender. Zipparo, M. (2026) https://BioRender.com/e8yc236.

While CD4 T cells are more commonly the focus of studies investigating HIV, it is important to recognize the critical role of monocytes and macrophages during infection and latency. Both immune cells are susceptible to HIV and can serve as a reservoir, contributing to HIV persistence in tissues [2,62,63]. As with CD4 T cells, initial HIV infection, active replication and latency all result in distinct metabolic phenotypes in myeloid cells (Figure 2) [10,64].

HIV infection promotes a pro-inflammatory phenotype in monocytes, corresponding to GLUT1 expression. This has been reported in a few studies, which demonstrate that monocytes from PWH have increased GLUT1 expression compared to PWoH [65,66]. Utilizing a cohort of treatment-naïve PWH, one study found elevated GLUT1 expression compared to PWoH, as well as increased glucose uptake in intermediate monocytes. This study also highlighted differences found within the monocyte subsets, where non-classical and intermediate monocytes had the highest increase in GLUT1 expression and glucose uptake compared to classical monocytes [65]. An additional study demonstrated elevated GLUT1 expression in all monocyte subsets when comparing treatment-naïve and healthy controls, and GLUT1 expression was correlated with HIV-RNA levels in total monocytes and the intermediate subset. Interestingly, no differences were observed in this study in basal OXPHOS or glycolysis levels in unstimulated CD4 T cells, even with increased GLUT1 expression on monocytes [66]. Together, these studies demonstrate a significant increase in glucose metabolism in monocytes during active viral replication.

Previous studies have highlighted the importance of the polarized state of macrophages, and how this alters their susceptibility to many types of infection [10,33]. Unpolarized macrophages are known to be more susceptible to HIV infection compared to polarized macrophages, and polarization is associated with significant alterations to metabolism [10,67,68]. Pre-integration HIV infection steps are blocked in M1 phenotypes, while post-integration steps are blocked in M2 phenotypes [68]. While most studies use extreme pro-inflammatory or anti-inflammatory methods of skewing to look at infection in macrophages, some studies have shown a “sweet spot” for infection [10,67]. One study, specifically assessing urethral macrophages isolated from penile tissue from PWH, demonstrated that macrophages are more easily infected and more likely to persist when expressing an in-between profile of polarization. This profile was characterized by the expression of markers associated with both M1 and M2 phenotypes, including IL-1, IL-4, and CD206 [10,69]. This preferred polarization state of macrophages is likely related to a corresponding metabolic state that increases permissiveness of infection. Other studies have demonstrated that infected macrophages and macrophage cell lines exhibit higher levels of metabolic dysregulation due to HIV replication and viral protein interactions, with alterations in metabolites from glycolysis, the TCA cycle, PPP, and glutamate metabolism, compared to uninfected macrophages [11,64,70,71,72,73]. One such metabolite, Hexokinase 1 (HK1), a glycolytic enzyme that commits glucose to glycolysis, has been shown to play a critical role in promoting the survival of HIV-infected macrophages in vitro [73]. HK1 helps to maintain mitochondrial membrane potential by binding to the membrane, and disruption of this binding can result in cytochrome-c release and subsequent macrophage apoptosis [73,74]. This study utilized viral reactivation in the U1 cell line as a model for replication, and demonstrated that reactivation of HIV with PMA resulted in increased HK1 expression, but not always increased HK1 activity [73]. HIV protein Vpr, when overexpressed, was found to facilitate HK1 association with the mitochondria of activated U1 cells [73]. Disruption of HK1 binding resulted in increased caspase 3 and 7 activity, and subsequent cell death, suggesting that viral proteins actively contribute to the survival of infected macrophages [73]. However, it is important to note that most of this data is obtained via in vitro work, and in vivo studies could yield different results. Together, these studies highlight how HIV infection and replication appear to alter macrophage metabolism, but further in vivo work is needed to truly understand these mechanisms and how the exact shifts in metabolism occur.

### 4.2. ART-Suppressed Infection

As monocytes are typically short-lived in circulation, very few studies have characterized metabolism in monocytes during HIV latency. However, the two studies described above also assessed vsPWH in addition to treatment-naïve PWH and PWoH. One study observed that vsPWH maintained elevated GLUT1 in monocytes compared to PWoH, with statistically elevated levels of GLUT1 and increased glucose uptake in intermediate monocytes [65]. In the second study, which also assessed immunological responders following ART initiation (IR, CD4 count > 350 cells/ul) and immunological non-responders following ART initiation (INR, CD4 count < 200 cells/ul), demonstrated that INR had higher expression of GLUT1 compared to PWoH on non-classical monocytes, but this difference was not observed with IR [66].

Shifts in macrophage metabolism in latency have also been characterized, though primarily through in vitro methods as they are difficult to access in vivo [10,11,51,70,75]. In a study utilizing a latently infected microglia cell line (HC69), suppression of glycolysis was observed compared to an uninfected cell line (C20) [51]. This study demonstrated that compared to latently infected CD4 T cells, the HC69 cell line more heavily relied on glutamate metabolism to feed into the TCA cycle for ATP output [51]. Similar to CD4 T cells, latently infected myeloid cells also relied on the PPP for NADPH and antioxidant networks [51]. An ex vivo study assessing urethral macrophages isolated from vsPWH yielded similar results. This study demonstrated an increase in glycolytic activity and reservoir reactivation following treatment with S100 calcium-binding protein A8 (S100A8), an alarmin produced by macrophages that increases glycolytic activity and mitochondrial respiration as measured by extracellular acidification rate (ECAR) and oxygen consumption rate (OCR) [76]. Treatment of urethral macrophages with S100A8 resulted in viral replication, but inhibition of glycolysis in these cells with 2-DG, prior to S100A8 treatment, stunted viral reservoir reactivation [76]. An in vitro study using primary monocyte-derived macrophages (MDMs) demonstrated that latently infected macrophages were also found to have increased metabolic dysfunction compared to uninfected macrophages [70]. Latently infected macrophages had increased events of mitochondrial fusion, and while all cells did not exhibit compromised mitochondria, they still experienced a reduced overall mitochondrial capacity as measured via OCR [70]. Macrophages with enlarged, fused mitochondria also experienced lipid droplet accumulation, which may be due to an accumulation of TCA metabolites from reduced and disrupted OXPHOS reliance [70]. In addition, these macrophages demonstrated flexibility in the preferred energy source required for survival and latency maintenance [70]. Specifically, utilization of glutamate, glutamine, and a-KG as energy sources to drive OXPHOS was uniquely used by latently infected macrophages, which has also been observed in other studies utilizing latent microglial cell lines [51,70]. However, in this study, it is important to note that latency was not induced by ART but rather defined by a lack of ongoing viral replication as measured via p24 presence in supernatant [70]. ART drugs themselves are known to impact metabolism in immune cells and should be taken into consideration in future macrophage-focused experiments. While these studies offer insight into metabolic alterations in latently infected macrophages, it is important to note that differences may be observed in vivo, resulting in potential gaps in our understanding of metabolism in latently infected myeloid cells.

## 5. HIV-Related Comorbidities Influenced by Alterations in Immunometabolism

**Figure 3:**
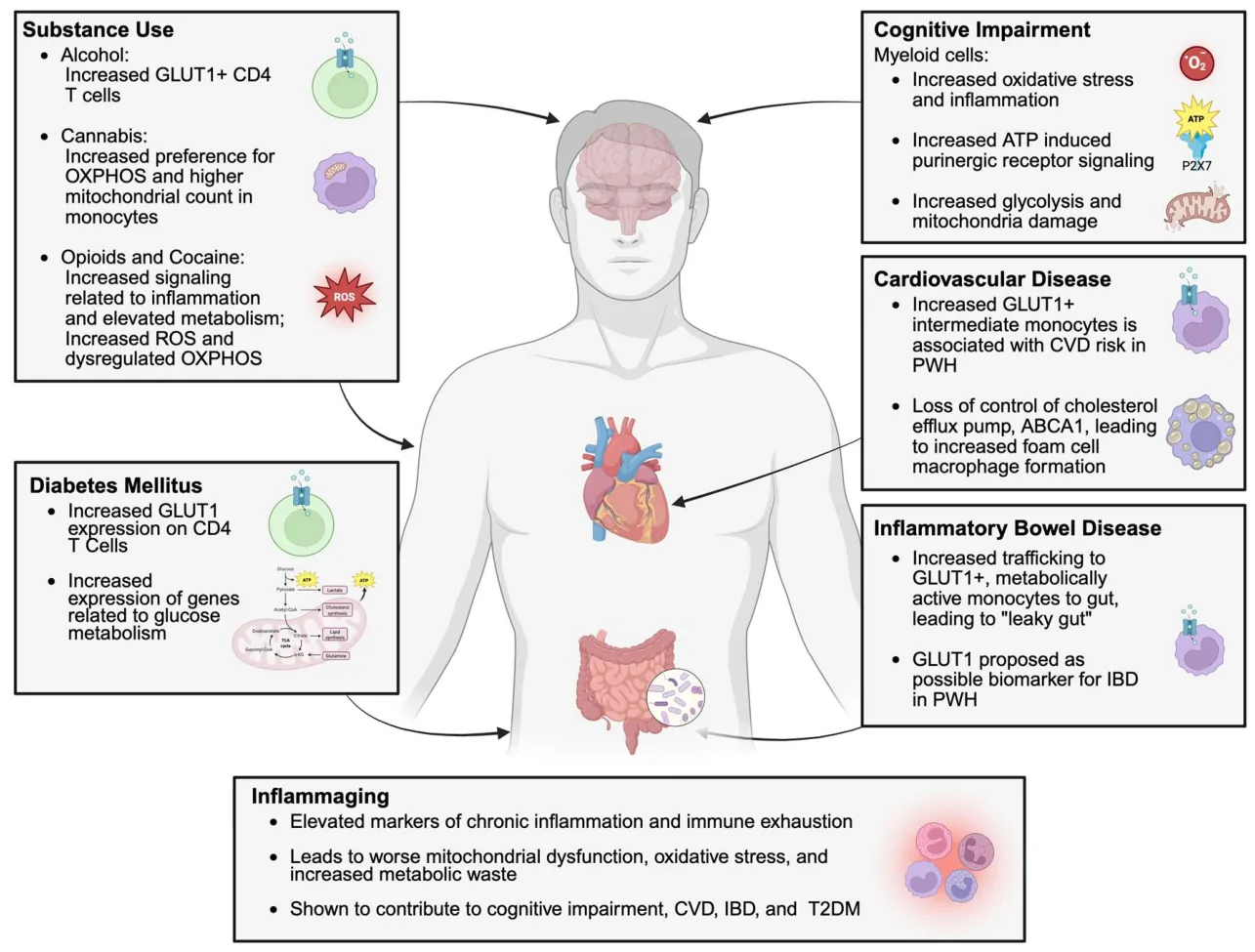
HIV-related comorbidities influenced by alterations in cell-specific immunometabolism. This figure highlights how shifts in cell-specific immunometabolism may contribute to HIV-related comorbidities, including substance use, diabetes mellitus, cognitive impairment, cardiovascular disease, inflammatory bowel disease, and inflammaging in general. Created in BioRender. Zipparo, M. (2026) https://BioRender.com/rqeeko9.

While ART has drastically improved the lives of PWH, it is not a cure [77], and PWH experience comorbidities at greater rates compared to PWoH despite successful viral suppression [6,7,10,11,48,78]. Evidence has consistently demonstrated that some of these comorbidities are driven, or further exacerbated, by immunometabolic dysfunction [11,48,78]. Many studies have highlighted broad drivers of immune–metabolic dysfunction, such as chronic inflammation, while few have assessed reservoir cell-specific changes in immunometabolism. The goal of this section is to highlight what is known at the cellular level and how cell-specific immunometabolism may contribute to relevant comorbidities and inflammaging in PWH (Figure 3).

### 5.1. Cognitive Impairment

Cognitive impairment, previously referred to as HIV-associated neurocognitive disorder (HAND), is prevalent in PWH and thought to be driven by ongoing neuroinflammation that leads to neurodegeneration. Under normal conditions, perivascular macrophages and microglia utilize OXPHOS for their metabolic requirements [79]. In the context of HIV, their metabolic profiles favor glycolysis, and this shift contributes to oxidative stress and mitochondrial dysfunction in the CNS, contributing to cognitive impairment [79]. MDMs from a cohort of PWH showed higher levels of pro-inflammatory cytokine profiles, lower levels of gene expression associated with mitochondrial biogenesis, and increased mitochondrial damage [80]. These combined factors likely contribute to neurodegeneration and cognitive impairment, as seen in greater global deficit scores compared to PWoH [80]. Recent studies have suggested that oxidative stress and purinergic signaling via extracellular ATP accumulation in myeloid cells further add to this comorbidity. P2X receptors, a type of purinergic receptor activated by ATP, can initiate critical immune responses, such as inflammation, play a key role in HIV infection and progression [48,81,82,83,84], and are expressed by various cell types within the central nervous system (CNS), including microglia, a known reservoir of HIV [48,85]. In vitro and in vivo work have shown that extracellular ATP, a potential biomarker for cognitive impairment, can be released as a result of HIV infection and interacts with the P2X7 receptor to promote neuroinflammation, likely contributing to cognitive impairment and neuronal cell death [48,83,86,87,88]. However, it is important to note that a majority of studies that explore the connection of P2X receptors in relation to cognitive impairment only look at the direct effects of viral proteins and not active replication or latency [83]. While heavily studied in the context of cognitive impairment, purinergic receptor signaling has been observed to impact other HIV-related comorbidities [78,79,84]. P2X receptors are also responsible for intracellular calcium regulation, a key component for many cellular pathways [83]. An increase in P2X signaling, leading to further inflammatory cytokine release and cellular stress, is suggested to increase risk for atherosclerosis, a CVD which is overrepresented in PWH [83,84,89]. Together, these studies demonstrate how altered metabolic pathways primarily within myeloid cells, coupled with shifts in purinergic receptor signaling in various anatomic compartments, may contribute to cognitive impairment in PWH.

### 5.2. Substance Use Disorders

Substance use disorders (SUDs) are prevalent among PWH and understanding how past and present use alters long-term immunometabolism is of significant interest [90]. While limited, recent research has highlighted the role of SUDs on immunometabolism of specific immune cells, with some studies focused on PWH. One study, utilizing CD4 T cells from healthy donors, found that ethanol (EtOH) exposure prompted increased CD4 T cell differentiation towards pro-inflammatory phenotypes, increasing Tbet (Th1) expression and decreasing FOX-P3 (Treg) expression [91]. This was accompanied by increased glycolytic activity in the EtOH-differentiated Th1 cells and decreased mitochondrial function in FOX-P3 cells [91]. As highlighted throughout this review, increased glycolytic activity in CD4 T cells is positively correlated with HIV infection and replication, potentially identifying a contributing factor of alcohol intake and HIV pathogenesis [3,4,91]. A recent study reported the impact of cannabis use on MDM immunometabolism. Monocytes were collected and differentiated from both PWH and PWoH [80]. In general, PWH who reported daily cannabis use were found to have monocytes with anti-inflammatory phenotypes, along with a preference for OXPHOS compared to glycolysis and a higher mitochondrial count, compared to PWH who had either never used or reported limited cannabis use [80]. This anti-inflammatory phenotype was also associated with better cognitive performance, suggesting that MDM immunometabolism may impact cognitive function in PWH [80]. While cannabis-induced immunometabolic shifts were found to have a neuroprotective effect, cocaine and opioids are reported to be more neurotoxic [92,93,94,95]. While there have been many studies that have assessed the effects of opioids on macrophage function, and some focused on global shifts in metabolism, only a few studies have assessed the effect of opioids on cell-specific metabolism in the context of HIV [96,97]. In general, opioids are known to impact cell signaling pathways, such as PI3k/Akt and mitogen-activated protein kinase (MAPK), that directly interact with cellular metabolism, and have been shown to facilitate HIV infection and replication [97]. Limited studies have focused on the intersection of cocaine, HIV, and cell-specific metabolism, but those that have are focused on the effects observed in the CNS [93,98]. One study, primarily utilizing glial cell lines, either infected with HIV or treated with HIV gp120, demonstrated increased AMPK expression after cocaine and HIV exposure, leading to increased ROS, dysregulated OXPHOS, and changes to the epigenetic landscape [98]. Interestingly, harnessing immunometabolism has been proposed as a way to treat neurotoxicity and inflammation in the context of cocaine use disorder (CUD) and HIV [93]. Treating primary rat cortical cell cultures with a cell-permeable form of itaconate, a TCA intermediate, combated the combined neurotoxic effects of HIV tat and cocaine, inhibiting pro-inflammatory cytokines and increasing protective antioxidant pathways [93]. However, it is important to note that these experiments were completed in vitro, utilized animal models or cell lines, and sometimes only treated with viral proteins. While these studies offer insight into SUD-induced cell-specific metabolic changes, future studies are needed to validate these findings in PWH.

### 5.3. Metabolic Diseases

As has been highlighted throughout this review, glycolytic pathways are among the most affected metabolic pathways in immune cells in PWH. Dysregulation of glucose metabolism has been highlighted as a factor driving many comorbidities, including CVD, inflammatory bowel disease (IBD), and type 2 diabetes (T2DM) [11,99,100,101,102]. Multiple studies of vsPWH have found an associated increase in GLUT1 expression on intermediate monocytes with the risk of CVD development [101,102]. In the case of inflammatory bowel disease (IBD), metabolically active monocytes (expressing elevated levels of GLUT1) traffic to the gut, leading to increased inflammation and “leaky gut” [99,103], resulting in GLUT1 expression being proposed as a potential biomarker for IBD in PWH [99]. Women with HIV diagnosed with T2DM have been reported to have elevated levels of GLUT1 expression, along with increased expression of genes involved in glucose metabolism, on CD4 T cells. However, following the initiation of anti-diabetic medication, some of these levels are normalized to those observed in women with HIV without T2DM [11,104]. In addition, CD4 T cells isolated from adipose tissue from PWH are more glucose intolerant, highlighting an increased risk of T2DM among PWH [11,105]. These studies demonstrate how glucose metabolism, specifically linked to increased GLUT1 expression on both CD4 T cells and monocytes, may be a suitable target to combat metabolic diseases in PWH.

Lipid metabolism in CD4 T cells and myeloid cells may also contribute to the progression of HIV-related comorbidities, specifically CVD. HIV infection and ART have been shown to alter lipid metabolism in PWH [106,107]. The observed changes in lipid profiles, coupled with chronic inflammation, likely influence the development of CVD and atherosclerosis [89,106,107]. Both HIV and simian immunodeficiency virus (SIV) accessory proteins, specifically Nef, have been shown to impair the transporter ABCA1 (ATP-binding cassette transporter A1), which controls cholesterol efflux in monocytes [89,108,109]. This impairment leads to increased foam cell formation, macrophages derived from infiltrating monocytes with excessive lipid droplet accumulation, which undergo cell death contributing to the necrotic core of atherosclerotic lesions and the overall progression of atherosclerosis [89,108,109]. Together, these studies highlight the direct impact of impaired lipid metabolism on the development of CVD and atherosclerosis in PWH.

### 5.4. Inflammaging

As ART has increased the life expectancy of PWH, new questions have emerged about how aging in PWH compares with that in PWoH [54,110]. PWH exhibit a more rapid immune-aging phenotype, or immunosenescence, driven in part by chronic inflammation (“inflammaging”) and immune exhaustion, as reflected by increased CD28− and CD57+ CD4 and CD8 T cells [54,111,112,113,114]. Both the natural aging process and the additional inflammaging observed in PWH have been shown to contribute to the metabolic diseases described above, including cognitive impairment, CVD, T2DM, and “leaky gut”/IBD [111,112,113]. The combined effects of inflammaging and immunosenescence in PWH also lead to broad mitochondrial dysfunction, mtDNA damage, oxidative stress, and the accumulation of metabolic waste, all of which contribute to metabolic disease [110,112,113]. Together, inflammaging, immunosenescence, and metabolic dysfunction at both the systemic and cellular levels may synergistically exacerbate cell-specific metabolic dysregulation in the comorbidities described.

## 6. Therapeutic Interventions to Target Immunometabolism

As highlighted throughout this review, the metabolic state of CD4 T cells and myeloid cells impacts their susceptibility to HIV infection and serves as a major determinant of latency [3,51,70]. For that reason, the concept of manipulating the metabolic state of reservoir-containing immune cells with targeted therapeutic interventions has been suggested as an HIV cure strategy and to treat comorbidities [115,116]. Both glycolysis and glutaminolysis are key metabolic pathways that are known to impact HIV infection and latency in reservoir-containing immune cells [3,51]. Previous studies have already highlighted how blocking these pathways results in reduced HIV infection and replication, and activation of these pathways promotes viral replication and inhibits latency [3,51]. While metabolic inhibitors as an approach for HIV cure are conceptually promising, both pathways are crucial for cell function and survival, and targeting them at the global level will likely pose a challenge [116].

The mTOR pathway, a master regulator of cell growth, metabolism, and survival, has been shown to be involved throughout HIV infection and latency, and targeting this pathway has been suggested as a potential cure strategy [40,55,115,116]. In a humanized mouse model, treatment with INK128, an mTOR inhibitor, resulted in decreased HIV viral loads, as well as demonstrated a suppression of HIV replication in both peripheral blood lymphocytes and a macrophage cell line [115,117]. Torin1 and pp242, two additional mTOR inhibitors, have been shown to suppress HIV reactivation in a latently infected CD4 cell line and CD4 T cells from PWH [55]. However, metformin, an indirect mTOR inhibitor typically used to treat T2DM, has been shown to have the opposite effect in PWH. Several studies have shown that metformin can lead to increased gene transcription and reactivation of viral transcripts in HIV-infected cell lines, as well as CD4 T cells from PWH [118,119]. One study reported that CD4 T cells from virally suppressed PWH treated with metformin were more likely to be recognized by broadly neutralizing anti-HIV-env antibodies compared to untreated CD4 T cells [118]. Suggesting that metformin induced greater Env expression on the surface of CD4 T cells. Given these opposing results, more research is needed to determine the specific aspects of the mTOR pathway that should be inhibited to alter HIV reactivation and latency in PWH.

Finally, nicotinamide adenine dinucleotide (NAD) targeted approaches have been proposed as a potential therapeutic to manage HIV replication and select comorbidities [120,121,122]. NAD, a crucial coenzyme involved in many metabolic and cellular functions, is known to be depleted during HIV infection [122]. Treatment with nicotinamide mononucleotide (NMN), a NAD precursor used to increase intracellular NAD, was found to suppress HIV viral protein production and replication, as well as partially inhibit CD4 T cell activation when treated with a latency reversing agent [122]. Additionally, in a humanized mouse model, NMN plus ART improved reconstitution of CD4 T cells compared to ART alone [122]. NMN has also been considered as a potential treatment to manage specific metabolic diseases [120,121]. HIV-Nef mice, a transgenic model where Nef is specifically expressed in the heart, treated with NMN resulted in decreased fibrosis of the heart and improved heart function compared to animals that were not treated [121]. A recent review highlighted the role of sirtuins (SIRTs) in HIV cognitive impairment. SIRTs are NAD+-dependent deacetylases and can be affected by Tat, promoting mitochondrial dysfunction and neuronal cell death to contribute to cognitive impairment. As NAD is depleted throughout the course of HIV infection, treatment with SIRT modulators, such as NAD+ precursors, can be used to activate SIRTs and promote neuroprotection [120]. As with all aforementioned therapeutic interventions in this section, further research is required to validate them using preclinical models and clinical trials. However, early evidence suggests that targeting metabolic pathways within reservoir-containing cells will likely advance HIV cure research and treatments for HIV-related comorbidities and should continue to be pursued.

## 7. Conclusions

As additional research continues to demonstrate the bidirectional relationship between HIV and immunometabolism, where HIV alters immune cell metabolism and immunometabolic changes influence HIV pathogenesis, it is becoming increasingly clear that the metabolic state of immune cells should be considered when attempting to decipher mechanisms of latency. The primary immune cell targets of HIV, including CD4 T cells, monocytes, and macrophages, undergo unique metabolic changes at each stage of the HIV life cycle. Emerging research has also highlighted the intersection of immunometabolism and HIV-related comorbidities, as underlying metabolic shifts can contribute to the development and persistence of these conditions, and while ART has revolutionized the lives of PWH, its effects on immunometabolism should be considered in future studies. Many studies have also suggested that manipulating immunometabolism may advance HIV cure efforts. While more research is needed in this emerging field, using metabolic reprogramming to target the latent reservoir and HIV-related comorbidities represents a promising avenue for future investigation.

## Data Availability

No new data were created or analyzed in this study. Data sharing is not applicable to this article.
